# Vestibular control of entorhinal cortex activity in spatial navigation

**DOI:** 10.3389/fnint.2014.00038

**Published:** 2014-06-05

**Authors:** Pierre-Yves Jacob, Bruno Poucet, Martine Liberge, Etienne Save, Francesca Sargolini

**Affiliations:** ^1^Laboratoire de Neurosciences Cognitives UMR7291, Fédération 3C FR3512, Université d’Aix-Marseille - CNRSMarseille, France; ^2^Institut Universitaire de FranceParis, France

**Keywords:** path integration, vestibular system, entorhinal cortex, grid cells, theta rhythm, rat

## Abstract

Navigation in rodents depends on both self-motion (idiothetic) and external (allothetic) information. Idiothetic information has a predominant role when allothetic information is absent or irrelevant. The vestibular system is a major source of idiothetic information in mammals. By integrating the signals generated by angular and linear accelerations during exploration, a rat is able to generate and update a vector pointing to its starting place and to perform accurate return. This navigation strategy, called path integration, has been shown to involve a network of brain structures. Among these structures, the entorhinal cortex (EC) may play a pivotal role as suggested by lesion and electrophysiological data. In particular, it has been recently discovered that some neurons in the medial EC display multiple firing fields producing a regular grid-like pattern across the environment. Such regular activity may arise from the integration of idiothetic information. This hypothesis would be strongly strengthened if it was shown that manipulation of vestibular information interferes with grid cell activity. In the present paper we review neuroanatomical and functional evidence indicating that the vestibular system influences the activity of the brain network involved in spatial navigation. We also provide new data on the effects of reversible inactivation of the peripheral vestibular system on the EC theta rhythm. The main result is that tetrodotoxin (TTX) administration abolishes velocity-controlled theta oscillations in the EC, indicating that vestibular information is necessary for EC activity. Since recent data demonstrate that disruption of theta rhythm in the medial EC induces a disorganization of grid cell firing, our findings indicate that the integration of idiothetic information in the EC is essential to form a spatial representation of the environment.

## Vestibular system and spatial navigation

To successfully navigate in their environment, humans and other animals can rely on two types of sensory cues, allothetic cues (environmental, e.g., visual, olfactory and auditory) and idiothetic cues (self-motion, e.g., vestibular, somatosensory information, motor efference copy, and optic flow). Because in a cue-rich environment animals may use both types of cues, the importance of idiothetic cues in navigation is mostly revealed through the ability of mammals to navigate without help of any external references (Etienne et al., 1996; Etienne and Jeffery, 2004). Thus, in the absence of relevant visual information (e.g., in darkness) or familiar landmarks, idiothetic cues support a form of navigation called path integration. An animal that performs path integration in darkness is, in principle, able to return by a straight path to its departure point after a sinuous journey through the environment. It is assumed that along its journey, the animal computes and continuously updates a return (or homing) vector pointing to the departure point by measuring and integrating angular and linear self-motion accelerations (Mittlestaedt and Mittlestaedt, 1982; Benhamou, 1997). Path integration involves multiple motor and sensory cues, and therefore necessarily involves multiple brain regions. Over the last two decades, data have accumulated showing that path integration involves a large network of brain areas including cortical and subcortical structures (Etienne and Jeffery, 2004). But how idiothetic cues are conveyed and processed by these structures and how they contribute to spatial navigation remains unclear.

The vestibular system is a major source of idiothetic information in mammals. The vestibular apparatus detects both angular and linear accelerations of the head, and these signals are conveyed to the central vestibular nuclei (VNC) to build a neuronal representation of angular and linear head velocity. Damage to the peripheral vestibular system in the rat produces deficits in a large variety of spatial tasks, including navigation tasks based on idiothetic (Wallace et al., 2002; Zheng et al., 2009) or allothetic information (Semenov and Bures, 1989; Ossenkopp and Hargreaves, 1993; Stackman and Herbert, 2002; Baek et al., 2010), and object recognition tasks (Zheng et al., 2004; Besnard et al., 2012; reviews in Smith, 1997 and Smith et al. 2010). These data indicate that the vestibular signal influences several aspects of spatial cognition, thus implicating a functional interaction between the vestibular system and the brain network involved in spatial information processing and navigation. Both neuroanatomical and lesion studies support this hypothesis.

**Figure 1:**
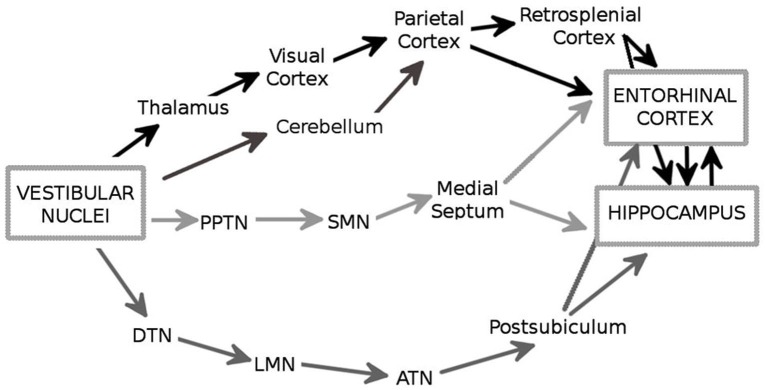
**The major anatomical pathways (in black, dark gray and light gray) through which the vestibular signals reach the hippocampus and the entorhinal cortex (EC)**. PPTN: pedunculopontine nucleus; SMN: supramammilary nucleus; DTN: dorsal tegmental nucleus; LMN: lateral mammilary nucleus; ATN: anterodorsal thalamic nucleus.

There are multiple ascending pathways that convey the vestibular signal to the cerebral cortical centers involved in spatial cognition, and particularly the hippocampal formation and the entorhinal cortex (EC; Figure 1; reviews, Smith, 1997; Smith et al., 2005; Shinder and Taube, 2010). The outputs from the VNC are conveyed by vestibulo-cortical pathways via the thalamus nuclei, i.e., lateral posterior, ventral posterior, medial geniculate, and ventrolateral geniculate nuclei, to several cortical regions including the visual and parietal cortices (Brotchie et al., 1995; Stackman et al., 2002). In addition, the vestibulo-cerebellar-cortical pathway conveys vestibular information to the parietal and retrosplenial cortices, which in turn project to the hippocampus through the EC (Amino et al., 2001 for a review see also Voogd et al., 1996). In this regard, it is interesting to note that the cerebellum influences hippocampal place cell activity when only self motion is used, e.g., in the dark (Rochefort et al., 2011). An important pathway involves the head-direction cell system, a network containing cells that are characterized by head direction-specific firing. The VNC fibers contact the dorsal tegmental nucleus which projects to the lateral mammillary nucleus, then to the anterodorsal thalamic nucleus and the postsubiculum, two structures that contain sharply tuned head-direction cells (Taube, 2007). This network eventually provides an input to the EC and to the hippocampus (Robertson and Kaitz, 1981; Witter et al., 1988; for a review see Amaral and Witter, 1989). Finally, it has been suggested that vestibular information reaches the hippocampus and the EC via the theta-generating system that involves the pedunculopontine tegmental nucleus, the supramammillary nucleus and the medial septum (Smith et al., 2005). Overall, the entorhinal-hippocampal system receives a large amount of highly processed vestibular information which may be essential for spatial information processing and navigation. Consistent with this hypothesis, several studies have shown that the vestibular signal has a strong impact on the activity of the different categories of spatially-selective cells. These neurons include the hippocampal place cells (O’Keefe and Dostrovsky, 1971), that fire when the animal moves through a particular location in space, and the head-direction cells in the dorsal presubiculum (or post-subiculum) (Rank, 1984) and the antero-dorsal thalamus (Taube, 1995), whose activity is controlled by the position of the head in space. Direct or indirect stimulation of the vestibular apparatus influences place cell activity. For exemple, Horii et al. (2004) showed that electrical stimulation of the VNC increased the firing rates of CA1 cells, in anesthetized rats. Similarly, Sharp et al. (1995) observed that indirect stimulation of the vestibular system through maze rotations strongly influenced the spatial properties of the hippocampal place cells. In addition, temporary and permanent inactivation of the peripheral vestibular system affects theta oscillations in the hippocampus (Russell et al., 2006) and disrupts location-specific firing of place cells (Stackman et al., 2002; Russell et al., 2003). Similarly, the direction-specific firing of head direction cells in the postsubiculum and the anterior thalamus is strongly altered following vestibular dysfunctions (Stackman and Taube, 1997; Stackman et al., 2002; Muir et al., 2009; Yoder and Taube, 2009). These results are in accordance with early studies showing that the firing properties of place cells and head-direction cells are influenced by idiothetic cues (Muller and Kubie, 1987; Wiener et al., 1995; Gothard et al., 1996; Rotenberg and Muller, 1997; Knierim et al., 1998; Save et al., 1998, 2000). Moreover, they demonstrate a major role of the vestibular system in controlling the firing properties of cells located in a brain network whose activity strongly supports spatial navigation.

Spatial signals are not exclusive to the hippocampal formation. Recent studies have shown that the EC, a major source of afferent input for the hippocampus (Steward and Scoville, 1976; Köhler, 1985; for a review see Amaral and Witter, 1989), contains both position-selective cells and head-direction cells (Fyhn et al., 2004; Hafting et al., 2005; Sargolini et al., 2006). The activity of this cell network is supposed to underlie self-motion based navigation. This hypothesis is supported by both lesion studies showing that damage extended to the entire EC or restricted to the medial portion impairs path integration in a homing task (Parron and Save, 2004; Van Cauter et al., 2013), and electrophysiological data showing the existence of spatially-selective cells in the same area. This last issue is further developed in the next paragraph.

## Entorhinal grid cells as a major support for navigation guided by self-motion cues

**Figure 2:**
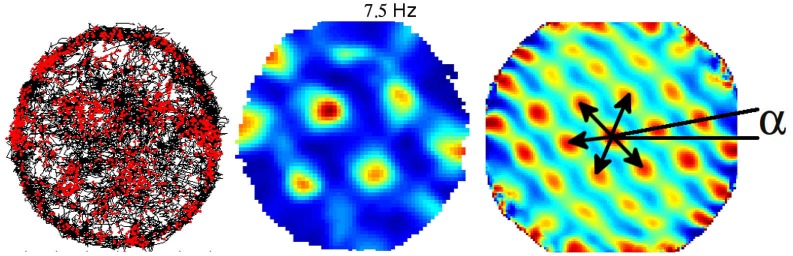
**Example of one grid cell recorded from the dorso medial entorhinal cortex (dMEC)**. Left: trajectory (black) with superposed spike locations (red dots). Middle: color-coded rate map with the peak rate indicated; red is the maximum firing rate, dark blue is zero, pixels not covered are white. Right: autocorrelation matrix of the rate map; the color scale is from blue (*r* = −1) through green (*r* = 0) to red (*r* = 1). Black arrows represent the distances between peaks (the scale of the map), and a represents the angle between the horizontal line and the first firing field array (the orientation of the map). The phase of the map is represented by the position of the firing fields.

Exploration of single cell spatial firing properties in the EC was initially motivated by the high density of inputs from the EC to the hippocampus. Following an initial report by Quirk et al. (1992) who recorded from the most ventral region, studies of the spatial firing properties in the EC culminated in the discovery of “grid cells” in the most dorsal region (Fyhn et al., 2004; Hafting et al., 2005). Grid cells share with place cells a form of location-specific firing but differ in that they are characterized by multiple firing fields arranged in a remarkably regular lattice of equilateral triangles (or regular hexagons). The activity of a given grid cell can be described with three parameters, namely, the scale, the orientation and spatial phase (Figure 2). Scale is represented by the field spacing, i.e., the distance between two vertices of the smallest triangle. Orientation can be taken as the angle in the range ±30° between horizontal and the closest triangle leg. Spatial phase is the location of a single reference field (Figure 2). Neighboring grid cells have similar orientation and scale but their spatial phases are distributed so as to cover the entire apparatus. The scale of the grid increases progressively as one gets more ventrally in the EC, and this property seems to depend upon the intrinsic oscillatory characteristics of the cells located at different levels along the dorso-ventral axis (Giocomo et al., 2007). The combination of grids at variable scales either within the EC or downstream in the hippocampus potentially provides a high resolution spatial coordinated system for navigation over large space. Grid cells are found mainly in layer II of the dorsal medial entorhinal cortex (dMEC), which projects to the dentate gyrus and CA3. In all other layers, grid cells coexist with head-direction cells and “conjunctive cells”, so named because their activity depends on head direction as well as location (Sargolini et al., 2006; Boccara et al., 2010), and border cells (Solstad et al., 2008). Altogether these cells may provide a conjunctive representation of position and direction as well as landmark-related information, thus providing a continuous update of the rat’s localization by self-motion cues and at the same time preventing cumulative drift during path integration.

The relative angles and densities of the vertices within grids of neighboring cells remain invariant both across environments and in response to changes in environmental cues. For example, changing the geometry of the environment (square vs. circle) does not alter the metric properties of the grid cell map in a stable manner: the scale and orientation of the map remain constant, whereas the spatial phase shifts randomly (Fyhn et al., 2007). Moreover, the grid map is stable in the absence of external landmarks (for example in the dark) (Hafting et al., 2005). Altogether these results indicate a strong dependence of grid cell activity on idiothetic cues. In essence, the metric organization of grid cell firing fields provides a directionally oriented, topographically organized neural map of the environment and the varying grid scale permits path integration calculations based on self-motion information (McNaughton et al., 2006). Recent studies however raise the question of whether such striking triangular organization is exclusively dependent on self-motion cues. When animals are exposed to a novel environment the grid map tends to expand (i.e., field spacing increases) (Barry et al., 2012; Stensola et al., 2012). A similar effect was observed following modification of the geometry of the environment (Barry et al., 2007). However, in both cases the grid map reverts to the original metric configuration following repeated exploration of the same environment, thus pleading in favor of the existence of an invariant self-motion-based spatial map (Jeffery and Burgess, 2006; Moser and Moser, 2008). It is presently unknown whether the grid cells provide such an invariant map and how external cues specifically influence their firing properties (Poucet et al., 2014). However there is no doubt that idiothetic cues do control grid cells’ activity and possibly contribute to the generation of their regular firing pattern. Thus, it is likely that the vestibular signal influences grid cell activity, but this issue has not been explored so far. To tackle this issue, we analyzed the effects induced by inactivating the vestibular system on the activity of the medial EC. We provide the first experimental evidence showing that the disruption of the vestibular signal induces a strong disorganization of the activity of the entorhinal neuronal network.

## Effects induced by vestibular inactivation on entorhinal cortex activity

In order to characterize how the vestibular signal influences EC activity, we studied the effects of temporary inactivation of the vestibular system on local field potentials (LFP) recorded within the dMEC. More specifically, we were interested in looking at the possible changes in oscillations in the theta band (5–12 Hz) within the dMEC neural network following disruption of the vestibular signal. According to recent models (Burgess et al., 2007) and experimental data (Brandon et al., 2011; Koenig et al., 2011), these oscillations are necessary to build the grid-like firing pattern. It is therefore likely that if vestibular inactivations disrupt theta oscillations in the dMEC, it would also result in a drastic disorganization of the grid-like firing pattern of the dMEC neurons.

**Figure 3:**
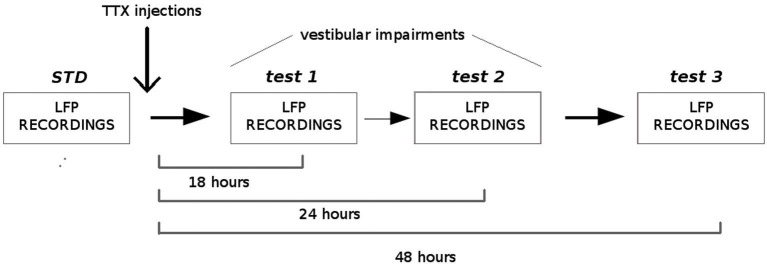
**Experimental protocol.** Local field potentials (LFPs) from the medial EC were recorded before and, 18 h, 24 h and 48 h after TTX administrations into the middle ear. Before each recording session, vestibular functions were assessed using two different tests: the “contact-righting” test and the “landing” test. All rats displayed vestibular impairments at both 18 h and 24 h delays. Vestibular functions were recovered 48 h after TTX injections.

Four adult Long-Evans rats (Janvier, France) were implanted with a bundle of four tetrodes aimed at the dMEC. The following coordinates were used, according to the stereotaxic atlas (Paxinos and Watson, 2004): AP: 0.6–0.8 mm anterior to the sinus, ML 4.8–5.0 mm from midline, DV 1.5 mm under the dura. After one week of recovery, the rats were trained to forage in two different enclosures (150 cm open arena and 150 cm circular track, 15 cm wide) to look for sugar pellets, and the electrodes were progressively lowered to reach the dMEC. When theta rhythm was detected in the LFPs, animals were submitted to the protocol described in Figure 3. Each rat was submitted to one-to-four successive recording sessions in each condition (STD (standard), test1, test2, test3), according to their level of exploration and locomotion. During the standard sessions (STD, number of sessions between 3 and 4 per rat, *N* = 19) LFPs were simultaneously recorded between two different tetrodes (one being the reference) at 1.024 Hz and filtered between 0.1 and 500 Hz (Hok et al., 2007). Immediately after the last recording session, the animals received a bilateral injection of 0.2 ml of tetrodotoxin (0.6 mM in PBS solution 0.1 M) into the middle ear through the tympanic membrane using a sterile needle (30 G) connected to a 10 ml Hamilton syringe, under general anesthesia (50% medetomidine 0.2 mg/kg and 50% ketamine 75 mg/kg). The rats were left in their home cage to recover from anesthesia. They were then submitted to three blocks of 20 min exploration sessions at 18 h (test 1, number of sessions between 1 and 2 per rat, *N* = 9), 24 h (test 2, number of sessions between 1 and 2 per rat, *N* = 9) and 48 h (test 3, number of sessions between 3 and 4 per rat, *N* = 13) after the TTX injection, and LFPs were recorded as previously described. At each delay, the vestibular dysfunction was assessed using the “landing” posture test and the contact-righting test. In the first test, the rat is lifted gently by the base of the tail. Intact rats extend their forelimbs toward the horizontal surface, whereas vestibular lesioned rats tend to curl their bodies ventrally around and towards their tail (Stackman and Herbert, 2002). In the contact-righting test the rat is placed supine on a tabletop surface and a Plexiglas surface is brought in contact with the ventral surface of the rat’s feet. Intact rats will rapidly right themselves whereas rats with vestibular impairments will “walk” about under the Plexiglas surface while in the supine position (Stackman and Taube, 1997). One rat was submitted twice to the entire protocol with a 30 days delay between the two TTX injections. Since no differences in both animal behavior and LFP recordings were observed between the two injections and between exploration of the different enclosures (open arena and circular track), all data were included in the statistical analysis. In order to characterize the oscillations in the theta band before and after TTX administrations, we calculated the Fast Fourier Transform of the power spectral densities and extracted power values in the theta band (5–12 Hz) and the delta band (1–4 Hz) (Matlab, FMA Toolbox distributed under General Public Licence, http://fmatoolbox.sourceforge.net/). We then expressed theta power as the ratio between the power spectrum in the theta and in the delta band. Finally we characterized the correlation between the peak frequency in the theta band and the velocity of the animal by calculating the Pearson correlation coefficient for 1000 ms intervals, for each recording session. The periods in which the rats were immobile or moved very slow (velocity less than 5 cm/s) were excluded from the analysis.

**Figure 4:**
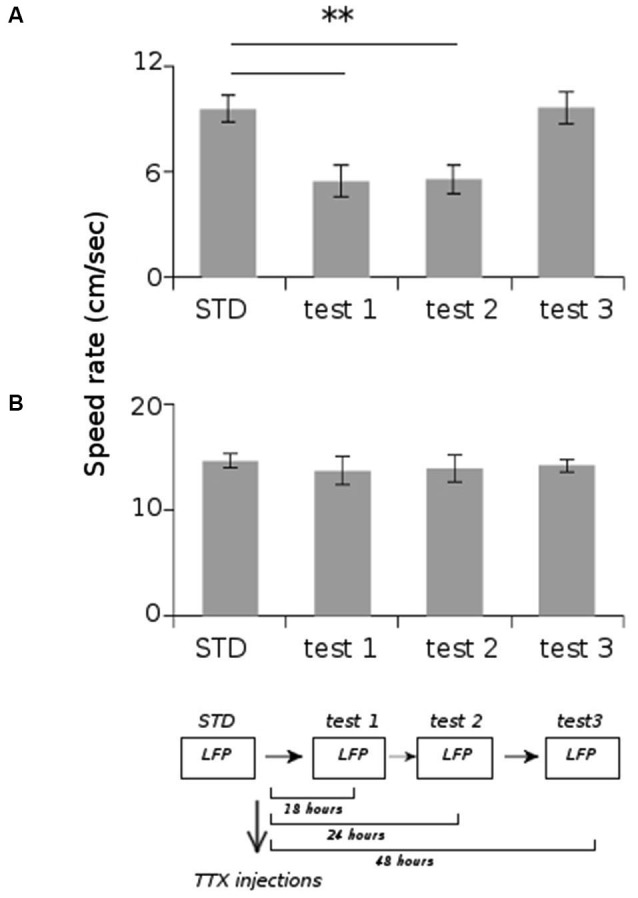
**Average locomotion speed rate of the rats during the different recording sessions**. **(A)** Total average speed rate. **(B)** Average speed rate excluding the periods of immobility (locomotion speed < 5 cm/s). ** *p* < 0.01, Tukey HSD test.

**Figure 5:**
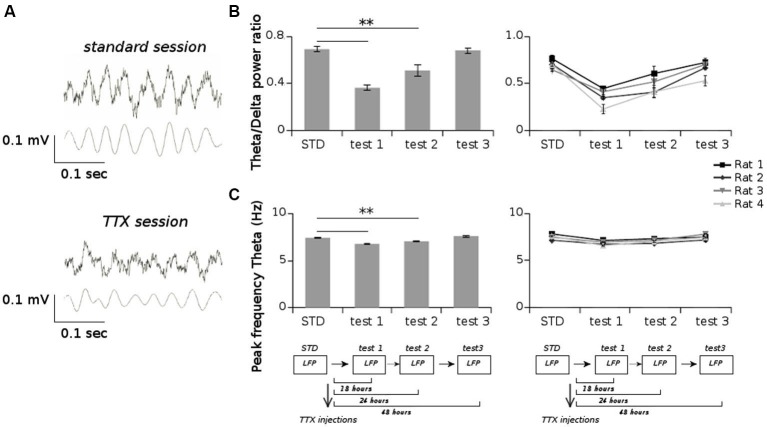
**(A)** Examples of raw LFPs (upper panels) and filtered LFPs in the theta band (5–12 Hz) (lower panels) from one session recorded before TTX injections (standard session) and one session recorded after TTX injections (TTX session). **(B)** Average values of the ratio of the power spectrum in the theta band (5–12 Hz) and in the delta band (1–4 Hz), for all sessions recorded before and after TTX administrations. **(C)** Average values of the peak frequency in the theta band for all sessions recorded before and after TTX administrations. ** p < 0.01, Tukey HSD test.

**Figure 6:**
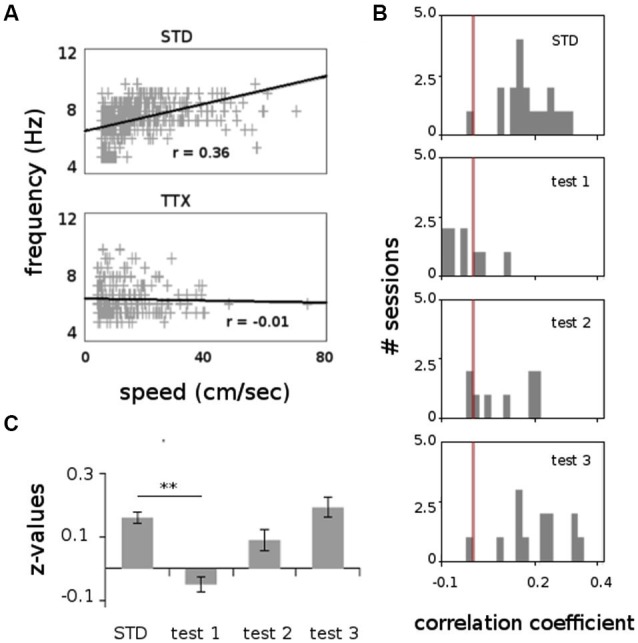
**Correlation between speed locomotion and theta frequency (1000 ms bins), before and after TTX injections. (A)** Scatterplot showing the relation between speed locomotion and theta frequency in two representative recording sessions before (STD) and during TTX administrations (TTX). Linear regression line and correlation values (Pearson’s product-moment correlation) are indicated. **(B)** Frequency distributions for the correlation values for all sessions recorded before and after TTX injections. The red bar represents the value *r* = 0. **(C)** Average values of the correlation coefficient (Fisher *z*-transformation) between speed locomotion and theta frequency. ** *p* < 0.01, Tukey HSD test.

All animals displayed strong vestibular impairments both at 18 h and 24 h, but vestibular function recovered 48 h after TTX injection, as assessed by the landing test and the contact-righting test. In addition, the rats exhibited typical behavioral symptoms of impaired vestibular function at both 18 h and 24 h delays, whereas such deficits were absent at 48 h delay condition. Such changes included head dorsiflexion, flattened posture with limbs adducted and rapid oscillatory head movements in the yaw plane. In addition, vestibular inactivation strongly decreased locomotion speed (Figure 4A; ANOVA delay effect *F*_(3,46)_ = 78.26, *P* < 0.01; Tukey Honestly Significant Difference (HSD), STD vs. test 1 *P* < 0.01, STD vs. test 2 *P* < 0.01). However, when all periods of immobility (locomotion speed < 5 cm/s) were excluded, no differences were observed (Figure 4B; ANOVA delay effect *F*_(3,46)_ = 2.08, *P* > 0.5). During vestibular inactivation we found a strong decrease of theta power (Figures 5A, B, left panel), and no effects on delta power (1–4 Hz normalized to the total power, *F*_(3,46)_ = 0.17, *P* > 0.5). A one-factor ANOVA showed a significant delay effect (*F*_(3,46)_ = 0.28, *P* < 0.01), which reflected the changes in theta/delta power ratio across the different time interval at which LFPs were recorded. Tukey HSD *post-hoc* analysis revealed a significant difference between STD sessions and both test 1 and test 2 (*P* < 0.01), but no difference with test 3 (*P* > 0.1). No significant difference was observed between rats (Figure 5B, right panel; Kruskal-Wallis ANOVA H(3, *N* = 16) = 2.50, *P* = 0.5) and a significant difference was observed between the different delay conditions (STD, test1, test2, test3; Kruskal-Wallis ANOVA H(3, *N* = 16) = 11.37, *P* = 0.009). A slight although significant reduction in theta peak frequency was also observed following TTX injection (Figure 5C; ANOVA: delay effect *F*_(3,46)_ = 1.47, *P* < 0.01; Tukey HSD, STD vs. test 1 *P* < 0.01, STD vs. test 2 *P* < 0.01 ). No significant difference was observed between rats (Figure 5C, right panel; Kruskal-Wallis ANOVA H(3, *N* = 16) = 3.20, *P* = 0.4) and a significant difference was observed between the different delay conditions (STD, test1, test2, test3; Kruskal-Wallis ANOVA H(3, *N* = 16) = 10.74, *P* = 0.013). Finally we calculated the correlation (Pearson’s product-moment correlation) between speed locomotion and theta frequency before and after vestibular inactivations. All periods of immobility were excluded from this analysis. Speed/theta frequency correlation was significant for the large majority of STD sessions (*P* < 0.05 in 16 sessions out of 19). On the contrary, during vestibular inactivations very few sessions showed a significant correlation value (test 1, total sessions *N* = 9, all non significant; test 2, *P* < 0.05 in 4 sessions out of 9). Forty-eight hours after TTX injection, speed-theta frequency correlation tended to be restored (test 3, *P* < 0.05 in 11 sessions out of 13; Figures 6A and 6B). The average values of the correlation coefficients at each delay condition confirmed this effect (Figure 6C). The one-factor ANOVA showed a significant delay effect (*F*_(3,46)_ = 15.42, *P* < 0.01). Tukey HSD post-hoc analysis revealed a significant difference between STD sessions and test 1 (*P* < 0.01), but no difference with test 2 and test 3 (*P* > 0.1).

Overall these results demonstrate that inactivations of the vestibular apparatus by trans-tympanic TTX injections provoke a strong decrease in power spectrum in the theta band and totally abolish the coupling between theta oscillations and locomotion speed. This indicates that the vestibular signal exerts a strong influence on the functional properties of the dMEC neural network.

## Conclusions

Path integration involves a large network of brain areas. Among these structures, the EC has been suggested to play a major role (McNaughton et al., 2006; Van Cauter et al., 2013). The discovery of grid cells, together with head-direction cells and conjunctive grid × head-direction cells, in the dorsal medial part of this structure strongly supports this hypothesis (Sargolini et al., 2006). The spatial activity of grid cells shows a relative independence of the environment and is maintained in spite of changes in running speed and running direction in darkness (Hafting et al., 2005). This suggests that vestibular signals mediating velocity and heading must be integrated over time to enable a constant representation of animal position. It is therefore likely that the vestibular system exerts a strong control over grid cell firing patterns. Here we show that vestibular inactivations strongly affect dMEC network activity by (1) decreasing the magnitude of theta oscillations; and (2) abolishing the correlation between velocity and theta frequency. The persistence of somewhat reduced theta rhythm indicates that such oscillations may be generated by the integration of non-vestibular inputs. However, the absence of theta-frequency speed dependence demonstrates that the vestibular signal is essential for velocity-controlled theta oscillations. Recent studies have shown that inactivations of the medial septum strongly reduce theta oscillations in the EC and abolish the grid-like firing pattern of the dMEC cells (Brandon et al., 2011; Koenig et al., 2011). This effect could be a consequence of the disruption of the velocity signal within the medial septum (King et al., 1998). Both the medial septum and the vestibular system may therefore control grid cell activity through modulation of velocity-controlled theta oscillations in the dMEC.

It is interesting to discuss these results in the frame of the theoretical models of grid field formation. The remarkable regularity of grid patterns has indeed inspired a large number of models over the last eight years. These models are generally divided in two categories, the oscillatory interference models and the attractor models. The first suggests that the firing pattern of the grid cells arises from temporal interference between an incoming theta rhythm and intrinsic membrane oscillations, such as those recorded by Giocomo et al. (2007) (Blair et al., 2007; Burgess et al., 2007; Burgess, 2008). The second postulates that the periodic grids is the result of interactions between local excitatory and inhibitory connections that creates a bump of activity which moves in correspondence with the rat’s motion (Fuhs and Touretzky, 2006; McNaughton et al., 2006; Burak and Fiete, 2009; Navratilova et al., 2012). Some models are based on both mechanisms (Hasselmo and Brandon, 2012), others discretize position into direction-dependent stripes or moduli (Gaussier et al., 2007) or small-scale grid networks (Blair et al., 2007) and use spatial interference to create a 2D hexagonal grid. In all cases, however, the velocity signal is essential for modifying and updating the spatial position (but see also Kropff and Treves, 2008). Presumably, such a signal arises primarily from the activity of the VNC. We speculate that inactivations of the vestibular system, which represents the primary input to the VNC, suppress the velocity signal (Angelaki and Dickman, 2000) and as a consequence disrupt the hexagonal pattern of grid cell activity. It should be noted, however, that vestibular impairments may be compensated at the central level by other inputs, such as those provided by the optic flow. Indeed optic flow is able to activate the VNC (Boyle et al., 1985; review in Cullen, 2012) and may provide a speed signal in the absence of vestibular signals. This could explain why in a virtual reality task functional grid cells are recorded and a speed-theta correlation is maintained despite the absence of vestibular motion signals (Domnisoru et al., 2013).

The vestibular system seems to exert a strong control over all categories of place-selective cells but it is unclear whether the mechanism is similar for all cell types. Vestibular lesions or inactivations provoke a drastic disorganization of the activity of place cells and possibly grid cells. In contrast, septal inactivations, which are supposed to disrupt the velocity signal, have a strong impact on grid cells but no effect on the spatial selectivity of place cells (although their firing frequency significantly decrease) (Koenig et al., 2011). In addition, Ravassard et al. (2013) have shown that in a cue-controlled virtual reality task functional place cells exist in the absence of theta frequency-speed dependence. This finding suggests either that the vestibular system influences the activity of place cells in a different manner compared to the grid cells (i.e., independently of its influence on the animal speed), or that different categories of place cells exist based on the sensory input that they receive. It is possible that some place cells are specifically controlled by self-motion cues and therefore influenced by the velocity signal, whereas other place cells are driven mainly by landmarks. This hypothesis could find a support on the observation that in a virtual reality task the percentage of active place cells is significantly lower than that observed in the real world (Ravassard et al., 2013). One may speculate that recording a greater number of place cells should reveal a small population of active place cells following vestibular inactivations.

In conclusion, the vestibular information potentially contributes to grid cell, place cell and head direction cell activity. These neurons form a network that combines self-motion cues and external cues to accurately track an animal’s movement through space. Characterizing multisensory integration processes within this neural network helps to understand the cooperation between these cells and the organization of spatial navigation process.

## Author contributions

Pierre-Yves Jacob and Francesca Sargolini planned the experiments. Pierre-Yves Jacob performed surgeries and LFP recordings. Martine Liberge and Pierre-Yves Jacob performed vestibular inactivations. Pierre-Yves Jacob and Francesca Sargolini performed data analysis. Pierre-Yves Jacob, Bruno Poucet, Etienne Save and Francesca Sargolini wrote the article. All authors participated to the discussion and the interpretation of the results.

## Animal research

All procedures complied with both European and French institutional guidelines (*Certificat n*°*A8/12/12, Ministère de l’Agriculture et de la Pêche*).

## Conflict of interest statement

The authors declare that the research was conducted in the absence of any commercial or financial relationships that could be construed as a potential conflict of interest.
